# Mis-splicing of the *GALNS* gene resulting from deep intronic mutations as a cause of Morquio a disease

**DOI:** 10.1186/s12881-018-0694-6

**Published:** 2018-10-11

**Authors:** Anna Caciotti, Rodolfo Tonin, Matthew Mort, David N. Cooper, Serena Gasperini, Miriam Rigoldi, Rossella Parini, Federica Deodato, Roberta Taurisano, Michelina Sibilio, Giancarlo Parenti, Renzo Guerrini, Amelia Morrone

**Affiliations:** 1 Molecular and Cell Biology Laboratory of Neurometabolic Diseases, Paediatric Neurology Unit and Laboratories, Neuroscience Department, Meyer Children’s Hospital, Viale Pieraccini n. 24, 50139 Florence, Italy; 2 Dipartimento di Neuroscienze, Psicologia, Area del Farmaco e Salute del Bambino, University of Florence, Florence, Italy; 3 Institute of Medical Genetics, School of Medicine, Cardiff University, Cardiff, UK; 4 Metabolic Unit, San Gerardo Hospital, Monza, Milan, Italy; 5 Division of Metabolism, Bambino Gesù Children’s Hospital, IRCCS, Rome, Italy; 6 Department of Translational Medical Sciences, Section of Pediatrics, Federico II University of Naples, Naples, Italy

**Keywords:** GALNS, Morquio a disease, Mucopolysaccharidosis IVA, Deep intronic mutations, mRNA defects, Whole gene sequencing

## Abstract

**Background:**

Mucopolysaccharidosis-IVA (Morquio A disease) is a lysosomal disorder in which the abnormal accumulation of keratan sulfate and chondroitin-6-sulfate is consequent to mutations in the galactosamine-6-sulfatase (*GALNS*) gene. Since standard DNA sequencing analysis fails to detect about 16% of *GALNS* mutant alleles, gross DNA rearrangement screening and uniparental disomy evaluation are required to complete the molecular diagnosis. Despite this, the second pathogenic *GALNS* allele generally remains unidentified in ~ 5% of Morquio-A disease patients.

**Methods:**

In an attempt to bridge the residual gap between clinical and molecular diagnosis, we performed an mRNA-based evaluation of three Morquio-A disease patients in whom the second mutant *GALNS* allele had not been identified. We also performed sequence analysis of the entire *GALNS* gene in two patients.

**Results:**

Different aberrant *GALNS* mRNA transcripts were characterized in each patient. Analysis of these transcripts then allowed the identification, in one patient, of a disease-causing deep intronic *GALNS* mutation. The aberrant mRNA products identified in the other two individuals resulted in partial exon loss. Despite sequencing the entire *GALNS* gene region in these patients, the identity of a single underlying pathological lesion could not be unequivocally determined. We postulate that a combination of multiple variants, acting in *cis*, may synergise in terms of their impact on the splicing machinery.

**Conclusions:**

We have identified *GALNS* variants located within deep intronic regions that have the potential to impact splicing. These findings have prompted us to incorporate mRNA analysis into our diagnostic flow procedure for the molecular analysis of Morquio A disease.

**Electronic supplementary material:**

The online version of this article (10.1186/s12881-018-0694-6) contains supplementary material, which is available to authorized users.

## Background

Mucopolysaccharidosis IV-A or Morquio A disease (MIM #253000] is an autosomal recessive lysosomal storage disease caused by the deficiency of *N*-acetylgalactosamine-6-sulfatase (GALNS), the lysosomal enzyme responsible for the hydrolytic degradation of keratan sulfate and chondroitin-6-sulfate [1]. GALNS is encoded by the *GALNS* gene (NM_000512.4) which is located on chromosome 16q24.3; the gene has a length of about 50 kb and is organized into 14 exons [2]. The *GALNS* gene is alternatively spliced, with two other reported protein coding transcripts in the RefSeq database (NM_001323543.1 and NM_001323544.1) [3]. In Ensembl, 13 transcripts are reported of which four are protein coding [4].

In excess of 330 different mutations have been reported in the *GALNS* gene causing Morquio A disease (Human Gene Mutation Database; http://www.hgmd.org). Among them, only one solitary deep intronic mutation which created a cryptic donor splice site was previously reported [5]. Empirically, standard sequencing procedures, covering all *GALNS* exons and intron/exon boundaries, have failed to identify ~ 16% of mutant alleles in patients affected by the disease [6, 7]. However, this percentage falls to 5% when gross DNA rearrangements are also screened for [8].

Morquio A disease affects multiple organ systems but its principal features are the cartilage defects, caused by keratan sulfate accumulation, that are responsible for the typical skeletal complications including coxa valga, scoliosis, short trunk dwarfism and cervical instability [1, 9]. The diagnosis tends to be particularly challenging in attenuated Morquio A patients, with consequent increased risk for missing the correct diagnosis [1].

Several therapeutic approaches including hematopoietic stem cell transplantation, gene therapy, substrate reduction therapy, and enzyme replacement therapy (ERT) have been developed in order to try to ameliorate the disease [10–14].

Newborn screening, whether by tandem mass spectrometry on dried blood spots and/or by fluorimetric assays, has proved to be both reliable and effective in identifying most mucopolysaccharidoses, including Morquio A disease [15, 16]. It is likely that the widespread adoption of this methodology will have a significant impact on the diagnosis of Morquio A disease [9, 17, 18].

Here we delineate the criteria that we have found efficacious in making an early diagnosis of Morquio A disease, together with a novel screening strategy that we have devised in order to optimize the probability of obtaining a molecular diagnosis in each case. Adoption of this strategy allowed the identification of novel splicing defects in three individuals in whom only one *GALNS* coding region mutation had originally been found.

## Methods

### Patients

The clinical features, and biochemical [urinary excretion of glycosaminoglycans (GAGs) and GALNS enzyme activity] and molecular analyses of these three Morquio A disease patients are summarized in Table 1. None of these patients has been previously reported. Based upon various empirical measurements to establish the severity of the MPS IVA phenotype [i.e. age at onset, growth/height (based on gender) and life span; [9, 19]], Pt1 and Pt3 are affected by the severe form of the disease, whereas Pt2 may be defined as mild.

**Table 1:** Clinical biochemical and molecular features of MPSIVA patients

Patient	1	2	3
Phenotype	severe	mild	severe
Age	14 y 6 m	16y	14y
Age at diagnosis	3y 5 m	8y	3y
Age at onset	3 m	3y	2-3y
GALNS activity (nmol/17 h/mg protein)	0.07 (nv 40–170)	0.2 (nv 40–170)	0 (nv 70–180)
Genotype	c.463G > A (ex 5)/ c.899-167A > G	c.463 G > A (ex 5)/ c.1002 + 307G > C^	c.697G > A (ex 7)/ c.759-67G > A^
mRNA alteration	−/ r.898_899ins53	−/ r.1003_1119del	−/ r.456_916del
Protein alteration	p.Gly155Arg / p.Gly300Valfs*37	p.Gly155Arg/ p.(Val335_Leu373del)	p.Asp233Asn/ p.(Lys153_Phe306del)
Sex	female	female	male
Parental consanguinity	no	no	no
Height (cm) at diagnosis	89.5	121	–
Height (cm) at last observation	108.6	136.3	114.5
Weight (kg)	18.2	26.6	27.5
Spondyloepiphyseal dysplasia	yes	yes	yes
Chest deformity	yes	yes	yes
Hearing loss	–	no	yes
Corneal clouding	no	mild	no
Coxa valga	yes	yes	yes
Odontoid hypoplasia	yes	yes	no
Hepatomegaly	no	yes	mild
Cardiomyopathy	yes	no	no
Valvular regurgitation	–	no	mitral/ tricuspid
Mental retardation	no	no	WISC-III TIQ 89
Surgery (type and age)	odontoid hypoplasia (7y), coxa valga (13y), cardiomyopathy (9 m)	right hip (6y), left hip (7y), coxa valga (10y)	coxa valga (10y)
Urine GAGs (mg/g creatinine)	92 (nv 48–82)	52 (nv 10–63)	6.6 (nv 2.1–23.2)

The patients’ parents gave their written consent for genetic testing to be performed on a local consent form, in accordance with the Declaration of Helsinki.

### Biochemical assays

Total urinary glycosaminoglycans (GAGs) were assayed as previously described [20]. Qualitative GAGs assay was performed by thin layer chromatography [21]. GALNS enzyme activity was measured from leukocytes/lymphocytes employing the fluorogenic method [22]. Beta-galactosidase (GLB1) activity was assayed in all patient samples as previously reported [23, 24] in order to allow exclusion of Morquio B syndrome.

It should be noted that control values for total urinary GAG evaluation can differ quite markedly between samples when the assays are performed in different centres, even though all such centres are qualified to perform the diagnostic assays. Urine keratan sulfate was found to be abundant in all samples from the three analysed patients.

### T-lymphocyte cell culture and treatment with cycloheximide to rescue aberrant transcripts from nonsense-mediated mRNA decay (NMD)

Patient and control T-lymphocytes were cultured in RPMI medium supplemented with fetal bovine serum (heat inactivated for 30 mins at 56 °C), interleukin 2 (800 U/ml), phytohemagglutinin (2.5 μg/ml) and antibiotics. The pool of control T-lymphocytes was separated from blood derived from 10 normal individuals. For each patient and control, lymphocytes were treated with (or without) 100 mg/ml cycloheximide (Sigma-Aldrich, Saint Louis, Missouri, USA) for 16 h. Total mRNA extraction and RT-PCR analysis are described below. Cycloheximide is used to identify cases of abnormal mRNA processing caused by mutations that generate premature termination codons (PTCs) that are then subject to NMD [25–27]. PTCs can result either from nonsense or frameshift mutations or from errors that occur during transcription or mRNA splicing [28]. Cycloheximide, being a translation elongation inhibitor, acts as a potent NMD inhibitor [26].

### Analysis of *GALNS* genomic DNA, total mRNA and cDNA synthesis

Genomic DNA was isolated from the patients’ peripheral blood lymphocytes. *GALNS* exons were PCR amplified using oligonucleotides and reaction conditions reported previously [8].

Isolation of total mRNA from cultured lymphocytes (with and without the addition of cycloheximide) was performed with the RNeasy Mini Kit (Qiagen, Hilden, Germany) for cells and tissues and the QIAamp RNA Blood Mini kit (Qiagen, Hilden, Germany) for blood samples. RNA concentrations were determined with a Nanodrop® ND-1000 Spectrophotometer (Nanodrop technologies, Wilmington, USA). RNA integrity was checked on a 1% agarose gel. *GALNS* mRNA reverse transcription was carried out as previously described [29]. Nucleotide numbering of the *GALNS* gene corresponded to the GenBank reference sequence, NM_000512.4. Patient mutational homozygosity and/or parental carrier status were verified by targeted DNA sequence analysis of the *GALNS* gene in each patient’s parents.

### Quantitative fluorescent PCR (QF-PCR) analysis and copy number variation (CNV) assays

QF-PCR fragments corresponding to all *GALNS* exons were obtained both by simplex and multiplex amplifications on genomic DNAs. CNV assays were performed combining TaqMan® MGB probe chemistry with Real Time PCR instruments (Applied Biosystems® 7500 Real-Time PCR) (Life Technologies Italia, Monza, Italy). The methods employed were as previously described [8].

### Whole *GALNS* gene sequencing and filtering of genetic variants

DNA samples from patients Pt2 and Pt3 were prepared using the TruSeq DNA PCR-Free protocol (Illumina Inc., San Diego, CA, USA) and sequenced with an Illumina HiSeqX Ten generating 150 bp paired-end reads. All fragments were mapped to the hg19 human genome reference sequence using iSAAC align version 03.16.12.05. Genetic variants identified within the *GALNS* gene region (NM_000512.4, chr16:88880141–88,923,374) in both patients (Pt2; 144 variants and Pt3; 73 variants) were filtered in an attempt to identify the variant(s) ultimately responsible for the observed splicing defects. The first filter was employed to eliminate variants called with low confidence (< 20 Phred quality score). A second filter was then applied to remove those variants which had been found to occur with a minor allele frequency of > 1% in the 1000 Genomes Project data (http://www.internationalgenome.org/). A location-based filter was also applied to remove those variants that were considered unlikely, based on their location, to be responsible for the splicing defect (thus, for example, the partial deletion of exon 10 in the aberrant mRNA transcript of Pt2 was deemed most unlikely to have been caused by a variant in exon 1). Therefore, for Pt2, this location-based filter was applied to the range chr16:88898406–88,891,277 (hg19), starting at the first base of *GALNS* IVS9 and extending to the last base of IVS10 (NM_000512.4). With patient Pt3, the location filter was applied to the range chr16: 88907400–88,893,246 (hg19) spanning the first base of *GALNS* IVS4 to the last base of IVS9 [the aberrant mRNA splicing phenotype in Pt3 involved the skipping of half of exon 5, all of exons 6, 7 and 8, plus half of exon 9]. Manual review of the remaining variants employed Human Splicing Finder 3.0 (HSF; http://www.umd.be/HSF3/) to assess the potential impact of these variants on the mRNA splicing phenotype. Zygosity in the patients’ parents was also employed to filter out likely non-pathogenic *GALNS* variants e.g. those variants found in the homozygous state in a clinically unaffected parent. The Combined Annotation-Dependent Depletion (CADD) method [30] was also used under manual review to rank the variants in terms of their potential pathogenicity; the higher the CADD score, the higher the probability that the variant is disease-causing.

## Results

### *GALNS* gene and mRNA molecular analyses

**Fig. 1:**
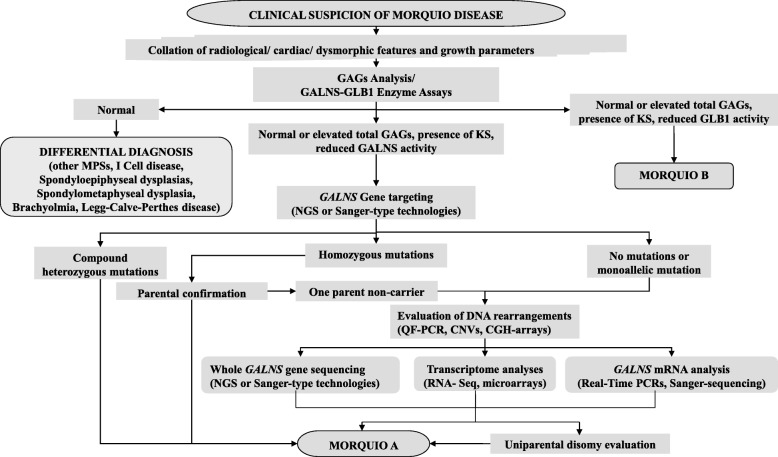
Diagnostic flowchart for Morquio A disease. The flowchart illustrates our clinical/laboratory analytical procedure. It should be noted that the procedure includes mRNA analysis in addition to DNA and protein analysis

In our cohort of about 40 Morquio A patients [8, 29, 31], the second disease-causing mutation remained uncharacterized in only three patients; the genetic analysis of these patients is reported here. Although heterozygous *GALNS* mutations were identified in all three patients (1, 2 and 3), the second anticipated pathogenic *GALNS* variant was not found in any of them by standard sequencing procedures. As depicted in the flowchart (Fig. 1), the presence of large deletions/duplications was excluded by means of quantitative fluorescent-PCR and copy number variation analysis as previously described [8].

### *GALNS* gene and mRNA molecular analyses in Pt1

Initial *GALNS* gene sequencing analysis of Pt1 revealed a paternally inherited heterozygous c.463G > A (p.Gly155Arg) mutation, previously described as being disease-causing [32], but no evidence of a maternally inherited lesion.

**Fig. 2:**
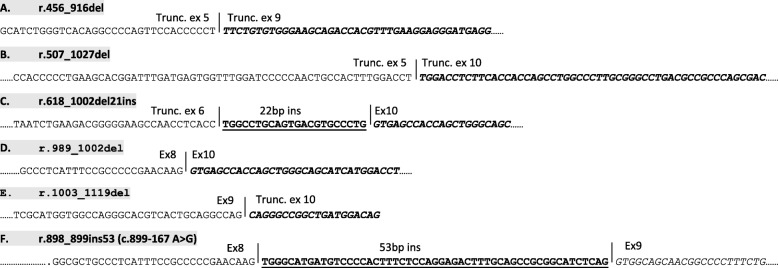
Aberrant splicing products detected in Morquio A patients in the heterozygous state. The alternative *GALNS* cDNA (RT-PCR) products were detected in samples from: **a** Pt3; **b**, **c** Mother of Pt3; **d** Pt3 and his father; **e** Pt2; **f** Pt1. Ten normal controls were included alongside the three patients in the mRNA analyses in order to compare the *GALNS* cDNA amplification products. The control group did not show any aberrant transcripts. Del, deletion; ins, insertion; trunc, truncated; ex, exon

*GALNS* mRNA analysis, performed by RT-PCR on lymphocytes from Pt1, revealed two distinct RT-PCR products, one corresponding to the wild-type *GALNS* transcript, the other slightly larger. The normal RT-PCR product harboured the c.463G > A (p.Gly155Arg) mutation whereas the aberrant RT-PCR product contained a 53-nucleotide sequence insertion between exons 8 and 9. When this inserted sequence was aligned to the *GALNS* gene sequence, it was identified as DNA sequence originating from intron 8, which had been included as a consequence of the activation of a cryptic donor splice site. Hence, the added DNA sequence constitutes, in effect, a cryptic exon (Fig. 2f).

The targeted sequencing of *GALNS* intron 8 from Pt1 then revealed the mRNA defect to be due to a novel heterozygous c.899–167 A > G transition that serves to create an additional donor splice site (Human Splicing Finder; http://www.umd.be/HSF3/). The upstream splice acceptor site that was co-activated, thereby allowing inclusion of the 53 bp cryptic exon, was an AG dinucleotide flanked by a stretch of pyrimidines on its 5′ side (Additional file 1: Figure S3). The sequence of the aberrant *GALNS* mRNA transcript predicted a frameshift ending in a premature stop codon located at position c.953 within exon 9 (Gly300Valfs*37) (Fig. 2f; Additional file 1: Figure S3). The c.899–167 A > G lesion was also detected in the heterozygous state in the mother of Pt1.

### The *GALNS* gene and mRNA molecular analyses in Pt2 and Pt3

Standard sequencing of the *GALNS* gene identified a single missense mutation in both Pt2 and Pt3 [p.Gly155Arg and p.Asp233Asn, respectively], but failed to identify the second disease-causing mutation in either patient. Further, RT-PCR analysis of the *GALNS* gene initially failed to reveal any additional RT-PCR products in Pt2 and Pt3 as compared to normal controls (data not shown). However, the same analysis performed on T-lymphocytes grown in the presence of cycloheximide revealed the presence of aberrant RT-PCR products in both patients (Fig. 2a-e).

The aberrant in-frame RT-PCR product detected in Pt2 would predict the omission of 117 nt from exon 10 of the *GALNS* transcript [r.1003_1119del, p.(Val335_Leu373del)] (Fig. 2e). The aberrant splicing product detected in lymphocyte cDNA samples from Pt3 was sequenced and shown to have resulted from the skipping of half of exon 5, all of exons 6, 7 and 8, plus half of exon 9 (r.456_916del; Fig. 2a) thereby generating a frameshift.

The *GALNS* mRNA evaluation performed in the father of Pt2 identified the same deletion, r.1003_1119del, that had been detected in the proband. By contrast, RT-PCR analysis revealed that the r.456_916del lesion detected in Pt3 was absent from his parents whilst two aberrant transcripts, characterized by different (albeit similar) deleted portions of *GALNS* exons, were detected in the mother of Pt3 (Fig. 2b and c). In addition, in both Pt3 and his father, an aberrant *GALNS* splicing product, that harboured the deletion of exon 9, was identified (Fig. 2d). In silico analysis suggested that both the c.697G > A (p.Asp233Asn) and c.775C > A (p.Arg259Arg) nucleotide changes could alter splicing (see HSF predictions given in Table 2); c.697G > A (p.Asp233Asn) in Pt3 corresponds to the pathological lesion harboured by the paternal *GALNS* allele. Since c.775C > A (p.Arg259Arg) was found *in cis* with the c.697G > A (p.Asp233Asn) mutation in Pt3 (i.e. it also has a paternal origin), it cannot correspond to the second pathological lesion anticipated in the *GALNS* gene of Pt3, despite its potential impact on splicing (Table 2). The c.697G > A (p.Asp233Asn) and c.775C > A (p.Arg259Arg) substitutions were nevertheless both identified, apparently in the homozygous state, in the aberrant splicing product lacking exon 9 that was detected in both Pt3 and his father (Fig. 2d), thereby confirming their likely role in generating this aberrant splicing product.

**Table 2:** *GALNS* nucleotide sequence variants with potential significance in the generation of the *GALNS* aberrant splicing products detected in Pt2 and Pt3

Chr: Pos (GRCh37.p13)	Zygosity	Parental Zygosity	Variant Type	Identifier (dbSNP 149, NCBI)	Exon/Intron	MAF	HSF Summary/CADD score
Pt2							
16:88898099	Heterozygous	Paternal (heterozygous)	c.1002 + 307G > C	rs866140272	INTRON 9	–	ESE gain and alterations to various ESRs - CADD 3.002
Pt3							
16:88901744	Heterozygous	Paternal (heterozygous)	c.775C > A; p.Arg259Arg	rs61742258	EXON 8	0.002	Cryptic donor splice site created, ESE loss and ESS gain - CADD 14.49
16:88901827	Heterozygous	Maternal (heterozygous)	c.759-67G > A	rs565875595	INTRON 7	0.001	ESE gain and ESS loss - CADD 6.652
16:88902194	Heterozygous	Paternal (heterozygous)	c.697G > A; p.Asp233Asn	rs753051547	EXON 7	–	Alteration of an exonic ESE site - CADD 24.1
16:88905035	Heterozygous	Maternal (heterozygous)	c.423-862C > T	–	INTRON 4	–	Cryptic donor splice site created - CADD 0.094

- = not present in gnomAD browser beta (http://gnomad.broadinstitute.org/); MAF = minor allele frequency; ESE = exonic splicing enhancer; ESS = exonic splicing silencer; ESRs = exonic splicing regulatory sequences; HSF (Human Splicing Finder) predictions = a tool which assesses the potential impact of these variants on the mRNA splicing phenotype (http://www.umd.be/HSF3/); CADD = Combined Annotation Dependent Depletion = a tool for scoring the deleteriousness of single nucleotide variants as well as insertion/deletion variants in the human genome. A CADD score of > 10 is applied as a threshold to identify high-confidence disease-causing mutations [27]

### Whole *GALNS* gene sequence analysis

Among the list of *GALNS* gene variants identified in genomic DNA samples from Pt2 and Pt3 [143 variants in Pt2 and 69 variants in Pt3, data not shown], in silico analyses identified several potentially pathogenic variants (summarised in Table 2).

The c.1002 + 307G > C variant detected in Pt2 was predicted to result in mis-splicing (Table 2) and represents a highly plausible candidate pathological lesion (i) on the basis of its location within intron 9, (ii) because the mutant allele creates an exonic splicing enhancer (ESE) site and (iii) because it is predicted to create an exon-identity element (ccgcct) [33] and may possibly also create or abolish splice silencer motifs [34].

The c.759-67G > A variant, identified in both patient Pt3 and his mother, represents the most plausible candidate for the observed splicing defect in these individuals. Although it should be appreciated that the consequences for mRNA splicing of changes in exonic splicing regulatory elements are often unpredictable, this deep intronic variant (c.759-67G > A) is predicted to abolish an existing exonic splicing silencer (ESS) motif while creating a novel ESE motif.

Intriguingly, the observed RNA splicing phenotypes differed between patient Pt3 and his mother. Thus, whereas the *GALNS* mRNA transcript in Pt3 harboured a 461 bp deletion (Fig. 2a), his mother exhibited two quite distinct mis-spliced transcripts, the first harbouring a 521 bp deletion, the second an indel comprising a 385 bp deletion together with a 21 bp insertion (Fig. 2b-c).

## Discussion

The spectrum of pathological mutations and benign polymorphisms in the *GALNS* gene displays considerable allelic heterogeneity [HGMD Professional (Stenson et al., 2017), Exome Variant Server (http://evs.gs.washington.edu/EVS/), GALNS Mutation Database (http://galns.mutdb.org/database) etc.]. However, in approximately 16% of patients, the anticipated second disease-causing *GALNS* mutation cannot be unequivocally identified within the gene coding region or at the exon-intron boundaries [6, 7].

Establishing a diagnostic plan, including the requisite genetic analyses, is essential to distinguish between bona fide Morquio A disease patients and individuals with other disorders presenting with similar clinical and radiological findings [6, 9, 12]. In addition, early diagnosis is crucial for the prompt deployment of available therapies before permanent systemic lesions occur. Here, we have integrated a series of clinical and analytical tools to provide a diagnostic flow chart for Morquio A disease (Fig. 1). In the algorithm we propose, next generation sequencing (NGS) procedures may be employed in two distinct analytical steps (Fig. 1). In the first, the exonic sequences and exon-intron boundaries of the gene in question are sequenced by NGS methodology (whole exome); alternatively, whole genome sequencing can be employed to sequence the entire gene region (~ 50 kb). In a second step, high-throughput sequencing-based methods can be used to perform transcriptome analysis (RNA-Seq) [35], including of course, in our case, *GALNS* transcripts.

We have presented here the cases of three Morquio A patients in whom the second disease-causing *GALNS* mutation was not initially identifiable either by standard sequencing procedures or by the analysis of gross DNA rearrangements and instead had to be determined by means of RT-PCR. Aberrant *GALNS* mRNA splicing products were noted in all three patients. In Pt1, the deep intronic mutation c.899–167 A > G was unequivocally identified as the lesion responsible for the aberrant mRNA (including an elongated exon) and a prematurely truncated GALNS protein. Hence, this aberrant splicing event can be directly and unambiguously related to the severe clinical phenotype observed in this patient.

Mutations located within deep intronic regions, that appear capable of promoting the use of alternative natural or non-natural splicing sites, were identified by *GALNS* whole gene sequencing analyses of Pt2 and Pt3 samples. After following the variant prioritisation protocol described above, specific *GALNS* variants in Pt2 (c.1002 + 307G > C) and Pt3 (c.759-67G > A) were predicted to make a contribution to the clinical phenotype in these individuals by impacting mRNA splicing. These variants exhibited a very low Minor allele frequency (MAF), and were predicted to modulate splicing, particularly with respect to potential ESE and ESS sequences.

Since both of the aberrantly spliced products detected in Pt2 and Pt3 disrupt exons, the mechanism responsible for these splicing alterations cannot be precisely ascertained. Indeed, it remains possible that a particular combination of variants could have been responsible for the observed splicing defects, rather than one variant on its own. Consequently, it may be that any of the other variants detected in the patients, including the putatively non-pathogenic *GALNS* variants (143 variants in Pt2 and 69 variants in Pt3, post-prioritization), may have contributed to the generation of the non-physiological splicing events detected e.g. c.423-862C > T identified in Pt3 and his mother. It should also be appreciated that variants such as c.423-862C > T may disrupt canonical splice junction sequences, i.e. cryptic acceptor and donor splice sites. Thus, it may well be that it is the combination of altered canonical and non-canonical splice sites in both patients that gives rise to these unique splicing alterations.

The *GALNS* gene is known to be alternatively spliced, with at least three known protein coding transcripts currently annotated (NM_00512.4, NM_001323544.1 and NM_00132354.1). It is possible that these alternative transcripts were differentially expressed between Pt3 and his mother; if so, this might have led to changes in splicing factor supply and demand, which could in turn account for the differences in the observed mRNA splicing phenotype between the patient and his mother.

Owing to the difficulties inherent in interpreting this atypical type of splicing event and our veritable ignorance of splicing regulatory regions, our approach did not unequivocally identify disease-causing variants at the DNA level for Pt2 and Pt3 even after intensive DNA sequence analysis of the *GALNS* gene region. Additional functional studies, including the construction of expression systems that variously combine the identified candidate mis-spliced variants, would be necessary to formally confirm our hypotheses. However, for diagnostic purposes, the pathogenicity of the observed mRNA splicing defects is evidenced by: a) the absence of RT-PCR amplification, corresponding to the aberrant mRNA transcripts detected in Pt2 and Pt3 samples, in a pool of 10 normal controls both treated and untreated with cycloheximide; b) the splicing products detected in Pt2 and Pt3 differing from the 13 known *GALNS* physiological mRNAs, collected in the Ensembl Human Genome browser (http://www.ensembl.org/index.html). These considerations imply that the alternative *GALNS* mRNA splicing products detected in Pt2 and Pt3 are non-physiological, and are therefore likely to be consequent to the (hitherto unidentified) second disease-causing *GALNS* alleles in these Morquio A patients.

## Conclusions

Morquio A disease is particularly prone to delayed diagnoses and/or misdiagnoses, owing to the difficulties inherent in the differential diagnosis of this rheumatic disease that requires specialist metabolic expertise. The addition of mRNA analysis and whole *GALNS* gene sequencing to this flowchart promises to help to identify those molecular causes of Morquio A disease which until now have been refractory to analysis. These analyses are likely to be particularly important for Morquio A screening programs in which the drawing up of a general diagnostic molecular plan is key to distinguishing between newborns who are carrying mutations associated with severe forms of the disease and those who are carrying mutations that are likely to give rise to milder or asymptomatic forms.

Our sequence analysis of the *GALNS* gene, involving gene level, genomic and RT-PCR analyses, suggests that although deep intronic mutations may be individually infrequent, they may be largely responsible for our occasional failure to identify *GALNS* disease alleles in Morquio A disease; it follows that marked improvements in our knowledge of the splicing machinery will be required before any diagnostic workflow can be regarded as being 100% effective.

## Additional file

Additional file 1 **Figure S3**. Sequence analysis of the aberrant mRNA transcript generated as a consequence of the c.899–167 A > G lesion identified in the *GALNS* gene of Pt1. **A.** *GALNS* mRNA sequence and schematic representation of the wild-type splicing event. **B.** Aberrant mRNA splicing resulting from the intronic c.899–167 A > G transition. (PPT 425 kb)

## Abbreviations

ERT: Enzyme replacement therapy
ESE: Exonic splicing enhancer
ESS: Exonic splicing silencer
GAGs: Glycosaminoglycans
GALNS: *N*-acetylgalactosamine-6-sulfatase
ISE: Intronic splicing enhancer
ISS: Intronic splicing silencers
MAF: Minor allele frequency
NGS: Next generation sequencing
NMD: Nonsense-mediated decay
