# Supplementary material for: Mis-splicing of the GALNS gene resulting from deep intronic mutations as a cause of Morquio a disease
Source: BMC Med Genet. 2018 Oct 11;19:183. doi: 10.1186/s12881-018-0694-6 (PMC6180571; doi:10.1186/s12881-018-0694-6)
Supplement: Supplementary file 1 — Figure S3. Sequence analysis of the aberrant mRNA transcript generated as a consequence of the c.899–167 A > G lesion identified in the GALNS gene of Pt1. A. GALNS mRNA sequence and schematic representation of the wild-type splicing event. B. Aberrant mRNA splicing resulting from the intronic c.899–167 A > G transition. (PPT 425 kb) [file 12881_2018_694_MOESM1_ESM.ppt]

## Slide 1
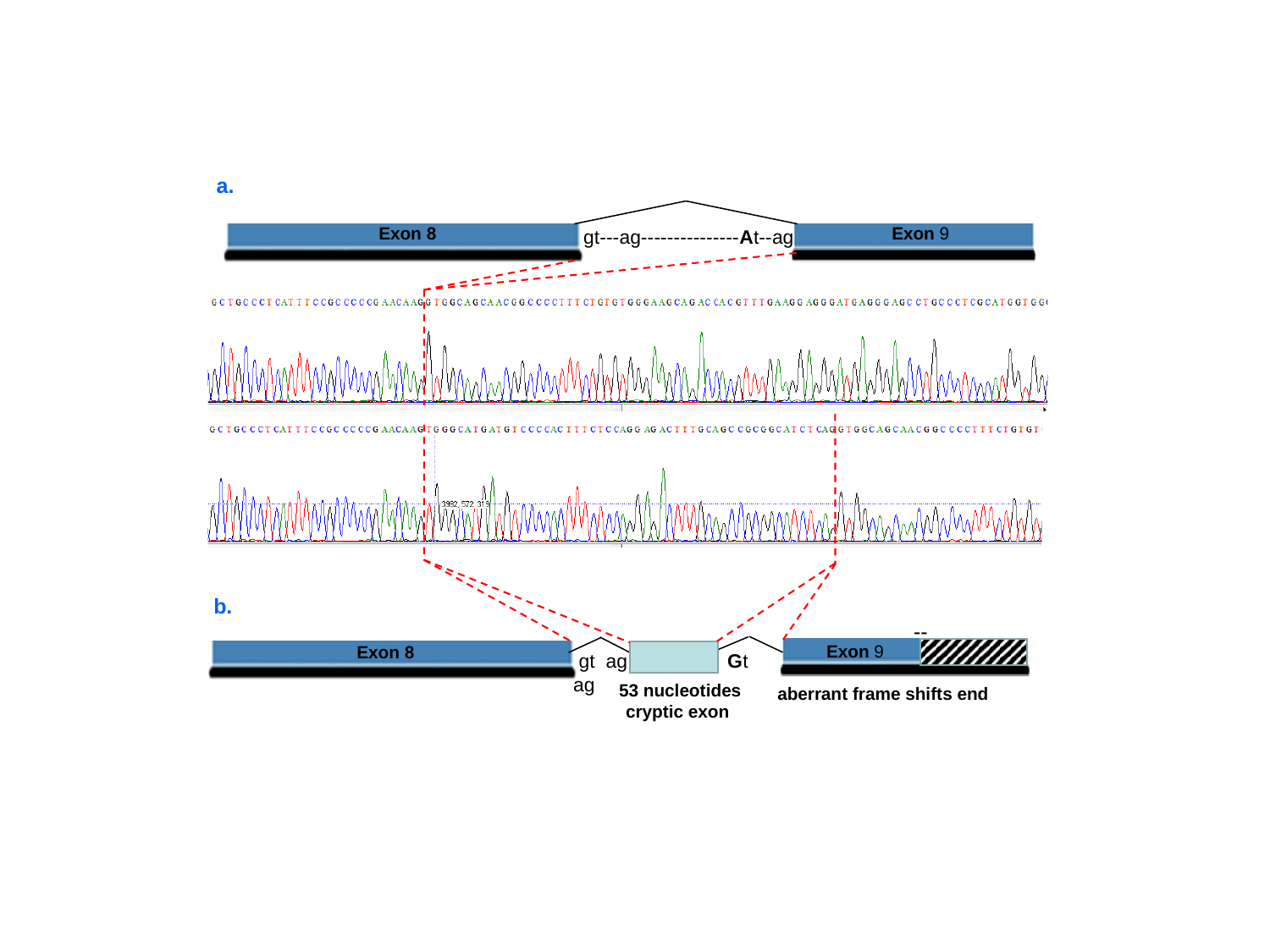

a.
Exon 8
Exon 9
gt---ag---------------At--ag
b.
--TGA--
Exon 9
Exon 8
 gt ag ------ Gt ag
53 nucleotides cryptic exon
aberrant frame shifts end
